# Unipolar Mania: Recent Updates and Review of the Literature

**DOI:** 10.1155/2014/261943

**Published:** 2014-04-30

**Authors:** Shubham Mehta

**Affiliations:** Department of Psychiatry, SMS Medical College, Jaipur Rajasthan 302004, India

## Abstract

*Introduction*. Unipolar mania (UM) has received less than the expected attention, when compared to its contemporary mood disorders, unipolar depression (UD) and bipolar disorder (BD). *Method*. The literature search included PUBMED and PSYCINFO databases. Cross-searches of key references were made to identify other articles of importance. *Results*. There seems to be a bipolar subgroup with a stable, unipolar recurrent manic course. Although UM does not have significant differences from bipolar mania in terms of sociodemographic variables, there are certain significant differences in clinical features. UM is reported to have more grandiosity, psychotic symptoms, and premorbid hyperthymic temperament, but less rapid cycling, suicidality, seasonality, and comorbid anxiety disorders. It seems to have a better course of illness with better social and professional adjustment. However, its response to lithium prophylaxis is found to be poor as compared to classical BD and valproate could be a better choice in this case. *Conclusion*. The available literature suggests that UM has certain differences from classical BD. The evidence, however, is insufficient to categorize it as separate diagnostic entity. However, considering UM as a course specifier of BD would be a reasonable step.

## 1. Introduction: Unipolar Mania—Then and Now

“Periodic mania” was the term which was first used by Kraepelin (1899) to refer to some of his cases having recurrent manic episodes without depression [1]. Wernicke (1900) proposed that single or recurrent episodes of mania or depression should be viewed as distinct disorders [2], separate from the ones which follow the continuous circular course of depression, mania, and free interval or “folie circulaire” as proposed by Falret [3]. “Phasic psychoses” were then classified by Kleist (1911, 1953) [4, 5] and Leonhard (1957) [6] who labeled pure mania and pure melancholia as “pure phasic psychoses” and manic-depressive psychosis as a “polymorphous phasic psychosis.” Genetic basis for distinction between unipolar mania and manic-depressive psychosis was first suggested by Neele (1949) [7].

The evolution of unipolar mania (UM) has continued since then, despite not receiving the distinct nosological status in the two most commonly used and accepted classificatory systems of DSM and ICD.

It did not find any place even in the recently introduced DSM-5 [8]. In the chapter of bipolar and related disorders, DSM-5 has clearly stated that the lifetime experience of major depressive episode is not a requirement for diagnosing bipolar I disorder. This implies that the isolated and recurrent episodes of mania also would fall into the category of bipolar I disorder.

ICD-10, however, has included “recurrent mania NOS” along with bipolar II disorder into the category of “other bipolar affective disorders” [9].

Thus, UM has received less expected attention than its contemporary mood disorders, unipolar depression (UD) and bipolar disorder (BD). Anyhow, it still manages to sparkle the nosological debate amongst researchers every now and then because there are a sufficient number of patients, reported from several countries and cultures, who demonstrate a recurrent unipolar manic course.

The paper reviews the available literature on unipolar mania. This would be of help to address the question that “whether unipolar mania stands apart as distinct nosological entity or not.” This also would serve to identify the gaps in the literature regarding UM which can guide the future research in this area.

## 2. Search Methodology

This update is based on the literature search carried out by the author. The literature search included PUBMED and PSYCINFO databases using the following keywords (in different combinations): “unipolar mania, recurrent mania, recurrent unipolar mania, periodic mania, and pure mania.” Cross-searches of key references were made to identify other articles of importance. No publication year limits were applied. The titles and abstracts were examined manually, and full-text articles of potentially relevant studies were obtained.

The available literature has been organized under the headings of prevalence, sociodemographic correlates, clinical features, laboratory investigations, and treatment issues and is discussed in comparison to bipolar mania at most places. Finally, the important findings are summarized and conclusions are made.

## 3. Prevalence

The prevalence of UM has ranged widely from as low as 1.1% [10] to as high as 65.3% [11] mainly because different defining criteria have been used by different researchers.

Perris in 1966 defined unipolar mania as “one or more manic episodes with no depressive episode” and found the prevalence of UM to be 4.5% among all bipolar patients [12]. This definition was continued to be used in most of the studies [10, 13–17] in 1970s and early 1980s which used retrospective chart reviews for their analysis. The prevalence of unipolar mania with this definition thus ranged from 1.1% of all bipolar patients [10] to 35.2% of bipolar inpatients [15]. Nurnberger et al. (1979), however, defined UM as minimum 1 hospitalization for manic episode and no hospitalization or somatic treatment for depression and found 15.7% of bipolar I disorder patients to be unipolar maniacs [18]. The retrospective studies which analyzed the prevalence of UM are shown in Table 1(a).

Srinivasan et al. (1982) defined unipolar mania as “3 or more episodes of mania without any depressive” and found its prevalence to be 40% in bipolar inpatients [19]. Since then, that is, 1990s and further, to label unipolar mania, the number of episodes was increased from 3 to 4 [20–23] or more and without any history of depression. The prevalence in these studies ranged from 16.3% [23] to 47.2% [22] among all bipolar patients. But the study design was still retrospective chart review (Table 1(a)). Also, there was no consensus on time duration. Aghanwa in 2001 defined unipolar mania as “3 episodes of mania or hypomania and at least 4 years of affective illness” [22] and Yazici et al. (2002) defined unipolar mania as “4 or more episodes of mania and at least 4 years of follow-up without any depressive episode” [23].

There had been three prospective studies which have reported the prevalence of UM (Table 1(b)).

Recently, a study by Andrade-Nascimento et al. in 2011 evaluated the differences between patients with manic episodes over the course of a 15-year illness duration compared to participants with histories of manic and depressive episodes and found that (5.6%) participants presented with unipolar mania (UM) [24]. Similarly, Perugi et al., in 2007, found that 21.8% of their patients in a 10-year illness duration were unipolar manic [25].

## 4. Sociodemographic Correlates

Most of the studies have not reported any significant difference in age of onset of unipolar mania over bipolar mania [13, 15, 19, 20, 22, 26]. A study on elderly unipolar and bipolar manic patients, however, found that elderly UM patients had an earlier onset [27]. Another recent study [11] reported earlier age of onset in unipolar mania (23 years) when compared to bipolar mania (28 years) which was almost similar to the findings of Yazici et al. (2002) [23].

Regarding gender, the results have been mixed with some early studies reporting a slight male preponderance [14, 28] and others finding no difference between both sexes [11, 15, 18, 20, 26, 29]. Furthermore, in other studies, UM was found to be more common in females than males [22, 23, 30].

Marital status [22, 23, 29], educational status [23, 26, 29], and occupational status [22, 29] do not differ in unipolar and bipolar mania.

The majority of studies on UM have come from “nonwestern” countries, such as Nigeria [26], India [20, 21, 29], the Fiji Islands [22], Turkey [23], Hong Kong [31], and Tunisia [11, 32], suggesting that UM is more common in these countries. But, due to lack of cross-cultural studies, this cannot be regarded as conclusive. However, a recent cross-cultural study reported that UM was three times more common in Tunisia than in France [32]. Two studies have been reported from USA. In these studies, the majority of the UM patients came from Iowa [15, 30] which was described as being a more rural setting than the other regions studied [30]. This, according to the authors, was the reason for the different prevalence of UM among various sites. The only study comparing the prevalence of UM among different ethnic groups, carried out in Fiji, found no significant differences in this regard [22]. Similarly, the ratios of white/black [14] or Caucasian/other patients [15] were not different in UM and bipolar mania groups. This probably refutes the possibility of difference in ethnicities being the reason for difference in prevalence of UM in different cultures. It would, however, be premature to propose an explanation based on the results of single study.

## 5. Clinical Features

Studies on clinical features have revealed some significant differences between UM and bipolar manias (Table 2).

Studies have also reported no differences in phenomenology and other clinical features between UM and bipolar disorders [19], number of episodes [22, 23, 29], duration of episodes [23], risk of psychiatric illness in first-degree relatives [11, 15, 18, 19, 22, 23, 27], and chronotype [29]. However, an Indian study by Avasthi et al. in 1996 noted that, out of 50 recurrent maniacs, 11 fulfilled the criteria for seasonal affective disorder and had onset in autumn [21], whereas another study by Mittal et al. (2013) reported spring and summer seasons to be periods of increased vulnerability for manic relapse [29]. Dakhlaoui et al. in 2008 reported the first episode season to be summer-autumn in UM [11].

With regard to family history, only Abrams et al. (1979) reported an increased risk of unipolar depression in the first-degree relatives of people diagnosed with UM [14], whereas Abrams and Taylor (1974) observed that unipolar maniacs had fewer relatives with affective illness, drug abuse, and characterological pathology compared to bipolar patients [13].

Other factors that may have a role in clinical presentation such as psychosocial variables, exposure to viruses, diet, and prenatal environment also should be taken into consideration in future studies [15].

## 6. Diagnostic Stability 

Among the studies, the duration of follow-up varies between 6 and 28 years. Nurnberger et al. (1979) reported that over a 4-month follow-up, 29% of the cases were reclassified as true bipolar disorder [18]. Perris (1982) observed that change in polarity from mania to depression mainly occurred by the third episode and rarely after the eighth episode [10]. Shulman and Tohen (1994) carried out a prospective chart review of elderly (>65 years) inpatient cohort. In their 27.7 years of follow-up, they did not find any change in polarity in any patient [27].  Table 3(a) summarizes the studies based on retrospective chart reviews which assessed the course of UM.

To date, there are three prospective studies which assessed the diagnostic stability of UM (Table 3(b)).

## 7. Laboratory Investigations

### 7.1. Neuroimaging

Neuroimaging revealed that UM patients had smaller third-ventricular width [33].

After brain injury, they had higher minimental scores and smaller subcortical, but larger cortical, lesions (primarily in right orbitofrontal and right basotemporal regions) than classical bipolar patients [34]. However, Cakir et al. (2008) found no differences in terms of neuropsychological tests between euthymic UM and BD patients [35].

### 7.2. Blood Chemistry

UM patients had less thyroid autoimmunity during chronic lithium treatment [36]. Pfohl et al. (1982) found significantly more abnormal blood count or chemistry in bipolar mania [15].

## 8. Treatment Issues

One of the most important findings in support of the view that UM is an entity distinct from BD is the difference in treatment response. Although no such difference has been reported with respect to the acute treatment of manic episode, different response characteristics to prophylactic treatment have been reported. The predominance of depression in BD patients has been associated with a better response to lithium maintenance therapy. Two studies compared the UM and BD patients in response to lithium prophylaxis (Table 4).

Husain et al. (1993) reported good response to maintenance ECT in case of an elderly female with recurrent unipolar mania (bipolar disorder, manic as per DSM-III) who was resistant to antipsychotics and mood stabilizers, intolerant to lithium, and treated with 81 ECTs over period of 2 years [37]. On the other hand, Angst et al. (2004) showed that BD-I is a heterogeneous entity. The “manic” group, namely, UM and BD-I patients with a marked preponderance of manic episodes during the course of illness, appeared to differ from the “classical bipolar” group in terms of some characteristics like lower risk of recurrence, chronicity, and suicide, better academic achievement, and longer duration of euthymia with or without maintenance treatment [38].

Recently, Yazici and Cakir (2012) [39] found that the response rate to lithium prophylaxis was significantly less in the UM group than that in the BD group, whereas the response rate to valproate prophylaxis was similar in both groups. These data suggest that valproate may be a better choice for prophylactic treatment in UM patients. Secondly, when the difference in the response to lithium prophylaxis was investigated, comparison of all the bipolar patients (UM and BD groups together) with a manic episode rate of <50% and >50% and <80% and >80% showed that the response rates were lower in the patients with manic preponderance and that the difference increased as the degree of preponderance increased; however, there was no difference when the UM group was excluded from the comparisons. These findings indicate that being less responsive to lithium prophylaxis was associated more closely with UM than with manic preponderance in bipolarity.

## 9. Summary 

The above review of literature clearly indicates that only a handful of studies pertaining to UM are available at present. The available literature shows that there has been no consensus regarding the definitions and diagnostic criteria of UM which has resulted in its prevalence ranging widely from 1.1% to 65.3%. The differences in study design (retrospective versus prospective) could be another factor which might have contributed to this. No differences have been found between UM and bipolar manias in most of the studies on sociodemographic variables like gender, age of onset of illness, marital status, educational status, and occupational status. UM appears to be more common in “nonwestern” countries, but there is substantial lack of cross-cultural studies to reach any firm conclusion. In clinical and/or psychopathological presentation, UM has more premorbid hyperthymic temperament, grandiosity, and psychotic symptoms but less rapid cycling, suicidality, comorbid anxiety disorder, and seasonality than bipolar mania. However, it is a clinically stable diagnosis over a period of time. It has also been reported that UM produces less social and work disability than BD. Regarding neuroimaging findings, UM shows significantly less third-ventricular size than bipolar mania but this awaits replication. As far as treatment is concerned, UM has poor response to lithium prophylaxis and valproate could be a better choice in these patients.

## 10. Conclusion

The evidence has thus accumulated in favor of UM over the time which indicates that this entity merits further study. There are certain issues which need to be explored and addressed in future. Firstly, there is a need to adopt stricter diagnostic criteria for UM. This would allow for a better interpretation of the data. Secondly, cross-cultural studies are needed to determine and compare the prevalence of UM in different cultures. Thirdly, although some differences in the clinical features of UM and bipolar manias have been found, these are not consistent across studies so as to act as indicators of a particular type of illness. Fourthly, differences in seasonality, neuroimaging, and treatment response point towards neurobiological differences between UM and bipolar manias which requires further inquiry and exploration.

Therefore, the available literature on UM at this point of time does not warrant it to be classified as a distinct disorder. Rather, adding UM as a course specifier to the diagnosis of BD would be a reasonable step to draw the attention of the researchers and guide them for future research in this area.

## Figures and Tables

**Table:** (a)

Author (year)	Prevalence	Definition
Perris (1966) [12]	4.5% among all BD patients	M ≥ 1, D = 0

Abrams and Taylor (1974) [13]	28% of BD I patients	M-number not defined, D = 0

Nurnberger et al. (1979) [18]	15.7% of BD I patients	M ≥ 1 hospitalization, D = no hospitalization or somatic treatment

Abrams et al. (1979) [14]	18% of BD patients	M ≥ 2, D = 0

Perris (1982) [10]	1.1% of BD patients	M ≥ 1, D = 0

Pfohl et al. (1982) [15]	35.2% of hospitalized BD patients	M ≥ 1, D = 0

Rao et al. (1982) [16]	2.7% of lithium clinic patients	Only M during follow-up, D = 0

Venkoba Rao and Madhavan (1983) [17]	12% of BD patients (age of onset >60 years)	Only M during follow-up, D = 0

Srinivasan et al. (1982) [19]	40% of hospitalized BD patients	M ≥ 3, D = 0

Margoob and Dutta (1988) [40]	42% of all BD patients	M = not defined, D = not defined

Khanna et al. (1992) [20]	44% of hospitalized BD patients	M ≥ 4, D = 0

Avasthi et al. (1996) [21]	6.45% of all affective disorders	M ≥ 3, D = 0

Aghanwa (2001) [22]	47.2% of all BD patients	M ≥ 3 (includes hypomania as well) and affective illness for at least 4 years, D = 0

Yazici et al. (2002) [23]	16.3% of BD I patients	M ≥ 4 and at least 4 years of follow-up, D = 0

Perugi et al. (2007) [25]	21.8% of hospitalized BD I patients	M ≥ 3 and affective illness of at least 10 years, D = 0

D: depressive episode; M: manic episode.

**Table:** (b)

Author (year)	Duration of follow-up	Prevalence	Definition
Makanjoula (1985) [26]	Five years	53% of manic patients	M ≥ 2, D = 0

Solomon et al. (2003) [30]	20 years	27 subjects had the diagnosis of unipolar mania at the time of entry. Seven of these did not suffer any depressive episodes during the 15- to 20-year follow-up.	Onset with M/HypoM, D = 0 for entire follow-up period

Dakhlaoui et al. (2008) [11]	Five years	65.3% of all bipolar I patients	M ≥ 2 and at least 5 years of follow-up, D = 0

D: depressive episode; M: manic episode; HypoM: hypomania.

**Table 2:** Differences in clinical variables between UM and bipolar mania.

Clinical variables	UM	Bipolar mania
Grandiosity [14, 15]	More	Less
Psychotic episodes [15]	More	Less
Psychotic symptoms [23]	More	Less
Psychotic first episode [23, 24]	More	Less
Congruent psychotic symptoms [15, 25]	More	Less
Rapid cycling [18, 23]	Less	More
Suicidality [18, 23]	Less	More
Comorbid anxiety disorders [24]	Less	More
Seasonality and seasonal problems [29]	Less	More
Social, familial, and work disability [24, 25]	Less	More
Marijuana and amphetamine [15]	More	Less
Hyperthymic temperament [23, 25]	More	Less

**Table:** (a)

Author (year)	Duration of illness (in years)	Comment
Abrams and Taylor (1974) [13]	10.86	—

Abrams et al. (1979) [14]	11.7	—

Nurnberger et al. (1979) [18]	—	29% of patients converted to BD over follow-up of 4 months.

Perris (1982) [10]	—	Polarity changes from mania to depression after 3rd episode and rare after 8th episode.

Srinivasan et al. (1982) [19]	5	—

Khanna et al. (1992) [20]	9.5	—

Avasthi et al. (1996) [21]	7	—

Aghanwa (2001) [22]	16.6	—

Yazici et al. (2002) [23]	12	—

**Table:** (b)

Author	Duration of follow-up	Findings
Makanjoula (1985) [26]	Five years	Only 13 out of 104 patients were found to suffer from bipolar disorder.

Solomon et al. (2003) [30]	20 years	Seven out of 27 (26%) patients continued to have diagnosis of unipolar mania.

Yazici et al. (2008) [41]	Seven years	30 out of initial 272 patients continued to be unipolar maniacs.

**Table 4:** Studies on UM assessing response to lithium prophylaxis.

Author	Findings	Conclusion (response to lithium prophylaxis)
Nurnberger et al. (1979) [18]	Response to lithium prophylaxis similar in patients with UM and BD hospitalized for depression; however, lithium is less effective in BD patients never hospitalized for depression.	UM < classical bipolar disorder

Yazici et al. (2002) [23]	Response to lithium prophylaxis similar in patients with UM and BD while using good, moderate, and poor response modes; however, when using responders and nonresponders as response mode, the UM group had significantly fewer responders than the BD.	UM < classical BD
